# Quantitative evaluation for spasticity of calf muscle after botulinum toxin injection in patients with cerebral palsy: a pilot study

**DOI:** 10.1186/s12984-016-0135-8

**Published:** 2016-03-12

**Authors:** Yu-Ching Lin, I-Ling Lin, Te-Feng Arthur Chou, Hsin-Min Lee

**Affiliations:** Department of Physical Medicine and Rehabilitation, College of Medicine, National Cheng Kung University, No.1, University Road, Tainan City, 701 Taiwan; Department of Physical Medicine and Rehabilitation, National Cheng Kung University Hospital, College of Medicine, National Cheng Kung University, No.1, University Road, Tainan City, 701 Taiwan; Medical Device Innovation Center, National Cheng Kung University, No.1, University Road, Tainan City, 701 Taiwan; Department of Medical Laboratory Sciences and Biotechnology, Kaohsiung Medical University, No 100, Shih-Chuan 1st Road, Kaohsiung City, 80708 Taiwan; School of Medicine, National Cheng Kung University, No.1, University Road, Tainan City, 701 Taiwan; Department of Physical Therapy, I-Shou University, No.8, Yida Rd., Jiaosu Village, Yanchao District, Kaohsiung City, 82445 Taiwan ROC

**Keywords:** Botulinum toxin, Cerebral palsy, Modified Tardieu scale, Spasticity, Quantitative measurement

## Abstract

**Background:**

Cerebral palsy (CP) is the most common pediatric disease to cause motor disability. Two common symptoms in CP are spasticity and contracture. If this occurred in the ankle plantar flexors of children with CP, it will impair their gait and active daily living profoundly. Most children with CP receive botulinum toxin type A (BoNT-A) injection to reduce muscle tone, but a knowledge gap exists in the understanding of changes of neural and non-neural components of spasticity after injection. The purpose of this study was to determine if our device for quantitative modified Tardieu approach (QMTA) is a valid method to assess spasticity of calf muscles after botulinum toxin injection.

**Methods:**

In this study, we intended to develop a device for quantitative measurement of spasticity in calf muscles based on the modified Tardieu scale (MTS) and techniques of biomedical engineering. Our QMTA measures the angular displacement and resistance of stretched joint with a device that is light, portable and can be operated similar to conventional approaches for MTS. The static (R2), dynamic (R1) and R2-R1 angles derived from the reactive signals collected by the miniature sensors are used to represent the non-neural and neural components of stretched spastic muscles. Four children with CP were recruited to assess the change in spasticity in their gastrocnemius muscles before and 4 weeks after BoNT-A injection.

**Results:**

A simulated ankle model validated the performance of our device in measuring joint displacement and estimating the angle of catch. Data from our participants with CP showed that R2 and R2-R1 improved significantly after BoNT-A administration. It indicates both neural and non-neural components of the spastic gastrocnemius muscles improved at four weeks after BoNT-A injection in children with CP.

**Conclusion:**

Our device for QMTA can objectively measure the changes in spasticity of the gastrocnemius muscle in children with cerebral palsy after BoNT-A injection.

## Background

Cerebral palsy (CP) is a group of permanent disorders attributed to a non-progressive insult to the developing fetal or infant brain [1, 2]. The type of brain damage observed in children with CP will lead to abnormal development of movement and posture which results in physical disability and limitations in activity [3–5]. Spastic type is the most common type of CP, which comprises about 80 % of reported cases [1, 2]. Although the brain injury caused by CP is non-progressive, the level of impairment related to spasticity on the musculoskeletal system is progressive and may result in deformity and contracture [3–5]. Long-lasting spasticity will inevitably change the biomechanical properties of muscles and soft tissues around the joints. Gradual deterioration of function such as ambulation may arise from changes in soft tissues, which is thought to be non-neural origin [6]. Among current therapies, botulinum toxin type A (BoNT-A) injection which blocks acetylcholine release at the neuromuscular junctions [7] has been one of the optimal options for children with spastic CP to reduce muscle tone of the lower leg and to improve the gait [8]. BoNT-A decreases spasticity at the level of body structure and function. It may lead to improved execution of walking activity and participation in daily life as demonstrated in improved levels of activity and participation based on the World Health Organization (WHO)’s international Classification of Functioning, Disability, and Health (ICF) [9, 10]. To comprehensively understand the effects of BoNT, from body function to activity and participation, further studies are required.

In this pilot study, BoNT-A injections were used to determine the sensitivity of our device to detect changes in spasticity. We want to know the effects of BoNT-A combined with rehabilitation programs on the neural and non-neural components of spastic muscles. However, there is only limited literature documenting these effects.

According to the definition of Lance [11], spasticity is a motor disorder characterized by a velocity-dependent increase in tonic stretch reflexes with exaggerated tendon jerks. Based on the consensus from the Task Force on Childhood Motor Disorders [12], spasticity is defined as one type of hypertonia in which one or both of the following signs are present: (1) resistance to externally imposed movement increases with increasing speed of stretch and varies with the direction of joint movement, and/or (2) resistance to externally imposed movement rises rapidly above a threshold speed or joint angle. The effect of BoNT on spasticity is most commonly evaluated with clinical scales such as modified Ashworth scale (MAS) [13] and modified Tardieu scale (MTS) [14, 15]. The MAS uses an ordinal scale to grade spasticity [16] and is the most common and convenient method to use in the clinical setting [17]. MAS assesses spasticity based on the resistance felt subjectively during passive stretch. However, the perceived resistance may be caused by neural and/or biomechanical origins, which MAS might fail to distinguish [18, 19]. In addition, the MAS has been criticized for their low reproducibility and poor accuracy in the previous review articles because it did not take into account the velocity-dependent aspect of spasticity [18, 19]. On the other hand, Boyd et al. developed the MTS as a clinical measurement for spasticity of lower extremities in children with cerebral palsy [14, 15]. The concepts used in MTS cover the velocity-dependent properties of spastic muscle as both slow and fast stretches are evaluated [20]. MTS utilizes manual stretch to measure the R2 and R1 angles of spastic muscle in the knee and ankle joint. R2 and R1 angles are defined as the first position that the examiner feels resistance when slow and fast stretch were applied to the joints respectively. R2 angle is generally accepted as the measurement of non-neural component of the stretch joint [14, 20]. In the neural component, the difference between R2 and R1 is more important than the individual values of R1. The dynamic component defined as R2 minus R1 (R2-R1), can be considered as the neural component of spastic muscles [20]. However, when measuring the angles of R1, R2 and R2-R1 with the universal goniometer, many factors such as stretched velocity and the reactive resistance during stretches may be overlooked. Furthermore, determining MTS angles with the goniometer might be imprecise due to errors of joint repositioning [21, 22]. If the joint displacement and reactive resistance can be measured quantitatively during MTS evaluation, then the response to BoNT treatment for spastic muscles may also potentially be quantified.

**Fig. 1:**
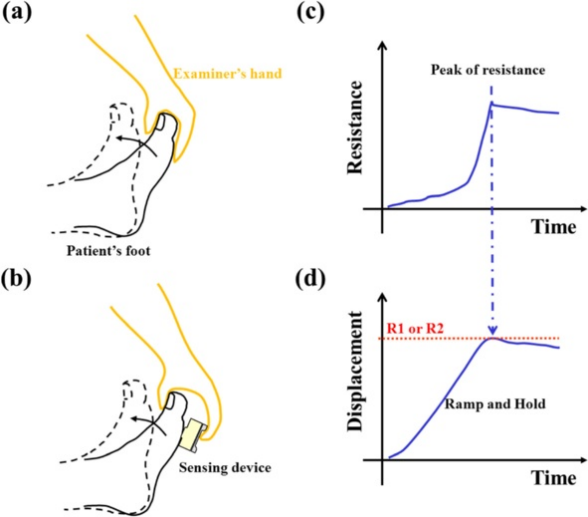
Rationales of spasticity measurement for modified Tardieu scale (MTS) and our quantitative modified Tardieu approach (QMTA). **a** Conventional MTS approach. **b** Our QMTA approach. **c** Expected curves of resistance during a manual stretch. **d** Expected curves of displacement during slow or fast stretches. Both curves of (**c**) and (**d**) are derived from a fast stretch on a spastic ankle joint of a patient with cerebral palsy

Quantitative MTS approach was first introduced by van den Noort et al. in 2009 [23]. They measured joint displacement by using commercial inertial sensors for manual stretch. The angle of catch (AOC) can be derived from the abrupt cessation of the joint displacement during fast stretch [22]. In recent years, Bar-On et al. have successfully used the MTS-like concept to develop an instrumented manual spasticity assessment for calf spasticity quantification [24, 25]. By using the combination of inertial and force sensors, the joint displacement and the reactive resistance can be recorded simultaneously during slow and fast stretch on the spastic muscle. The AOC was then derived from the joint displacement data according to the biomechanical or neurophysiological information such as angular deceleration, change of force or electromyography (EMG) signal. They found the MTS-like quantitative approach provides moderately high reliability and clinically relevant measures for calf spasticity [25]. Compared to motor-driven quantification approach [26, 27], the advantage of these manually-applied approach is the involvement of an experienced evaluator to improve patient compliance during the study. However, the traditional method using the instrumented manual approach requires the attachment of sensors on a device such as a dynamic orthosis around the joint [20, 25]. This may reduce patient compliance especially during evaluation of pediatric patients. Fixation of limbs around joints is unnecessary with our device. In our study, we tried to reduce the size of our sensors and apply it in a clinical manner we use every day in clinics (Fig. 1(a) and (b)). Furthermore, few studies have used the manually-applied quantitative approach to analyze the therapeutic reactions of BoNT injection on both neural and non-neural components of spastic calf muscles in patients with CP.

There were two main objectives of our study. First, we developed a hand-held device for objective measurement of calf muscle spasticity to improve record of angles with the traditional MTS. The measuring device was built to be used with the same manner and rationale of MTS, which we called quantitative modified Tardieu approach (QMTA). We also used a simulated ankle model to validate the recording of joint displacement and reactive resistance before applying it to children with CP. Second, we aimed to use QMTA in children with cerebral palsy to study the changes of neural and non-neural components of spastic calf muscle after BoNT-A injection. We hypothesize that both neural and non-neural components of the spastic calf muscle will improve at four weeks after BoNT-A injection by measuring the quantitative parameters with our QMTA.

## Methods

The rationale for modified Tardieu scale to determine the angles related to spasticity is illustrated in Fig. 1. In conventional MTS approach [14, 15], the examiner holds the upper part of the foot and utilizes a slow and fast stretch to assess the range of motion (ROM) for the ankle joint (Fig. 1 (a)). To differentiate the contributions of gastrocnemius and soleus for spasticity in calf muscles, we routinely test ROMs of ankle with the knee placed in flexion and extension. To examine the resistance of gastrocnemius, the hip and knee of participants were maintained in extension and the ankle joint was moved from maximal plantarflexion to maximal dorsiflexion. The same procedures were performed for testing the soleus, except the ipsilateral hip and knee were flexed at 90°. All four participants in our trial received BoNT-A injection to the gastrocnemius. The hips and knees of participants were maintained in extension when we performed the R1 and R2 examinations of ankle joints. The end ROM is determined when the examiner feels the resistance barrier of the joint, which can be defined as the peak resistance during stretch (Fig. 1 (c)). In traditional MTS setup, the examiners measure the displacement angle with a universal goniometer to find the R1 or R2 angle. Our QMTA is designed to place a sensing device between the patient’s foot and the examiner’s hand to record the resistance and the displacement during manual stretches (Fig. 1(b)). After determining the peak resistance, we can find the corresponding displacement angle of the ankle joint for R1 and R2 (as shown in Fig. 1(c)-(d)).

### System setup

**Fig. 2:**
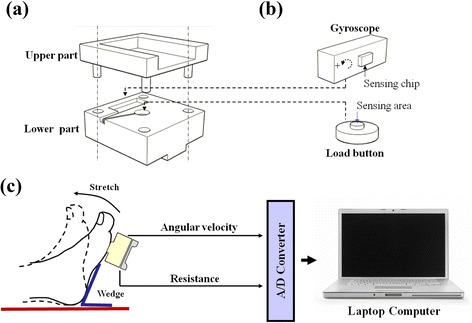
The spasticity measurement system for the quantitative modified Tardieu approach. **a** Mechanical parts of the sensing device. The upper and lower parts were connected via four sliding tracks to decrease the friction during compression. **b** A gyroscope was used to record angular velocity and was applied during ankle displacement to identify the angular displacement. A miniature load cell (load button) was used to record resistance during stretch. **c** The hand-held sensing device recorded and sent signals (angular velocity and resistance) to a computer via an A/D converter during ankle stretch

An integrated system with angular velocity and resistance measurement was developed to determine the static (R2) and dynamic (R1) angles of spasticity during manual stretches (as shown in Fig. 2). Details of the major components are described as following.

The device used for spasticity measurement is integrated as a hand-held device with dimensions of 5 cm × 5 cm × 3 cm (length x width x height) and with weight of 125 g (Fig. 2(a)). Upon stretching of the spastic muscles, the sensing device will be placed firmly in contact with the ball of foot and the angular velocity of that foot will be recorded along with the reactive resistance produced by the stretched foot. As showed in Fig. 2(a) and (b), a one-axis rate gyroscope module (2.5 cm × 1 cm × 1 cm; ADXRS300, ADI Co., Ltd) was implemented in the lower part of the device to sense the angular velocity of the stretched foot. By using the integration algorithm, the displacement of ankle joint can be derived from the angular velocity signal. Furthermore, a miniature load cell (1 cm diameter and 0.3 cm height; LBS-100, Interface Co., Ltd) was mounted at the center of the lower part. Since the sensing area of the load cell is higher than the surface of the lower part of the device, the reactive resistance from the stretched foot can be transmitted to the load cell and be recorded continuously.

As shown in Fig. 2(c), a laptop computer was used to administer the data acquisition and serve as the graphical user interface (GUI). The measured velocity and resistance signals were acquired by the computer through a 12-bit A/D converter (DAQCard-6036E, National Instruments Inc.) at a sampling rate of 100 Hz for storage and display of the data. A customized LabVIEW-based program (Version 7.1, NI, Inc.) with an integrated user interface provides the setting of parameters, display of data, and gathering of the signals during the experiment. Furthermore, the examiner can consult the computer screen in order to visualize the resistance and velocity of the applied stretch to help him/her maintain similar maneuvers among trials.

### Validation of measurement system

**Fig. 3:**
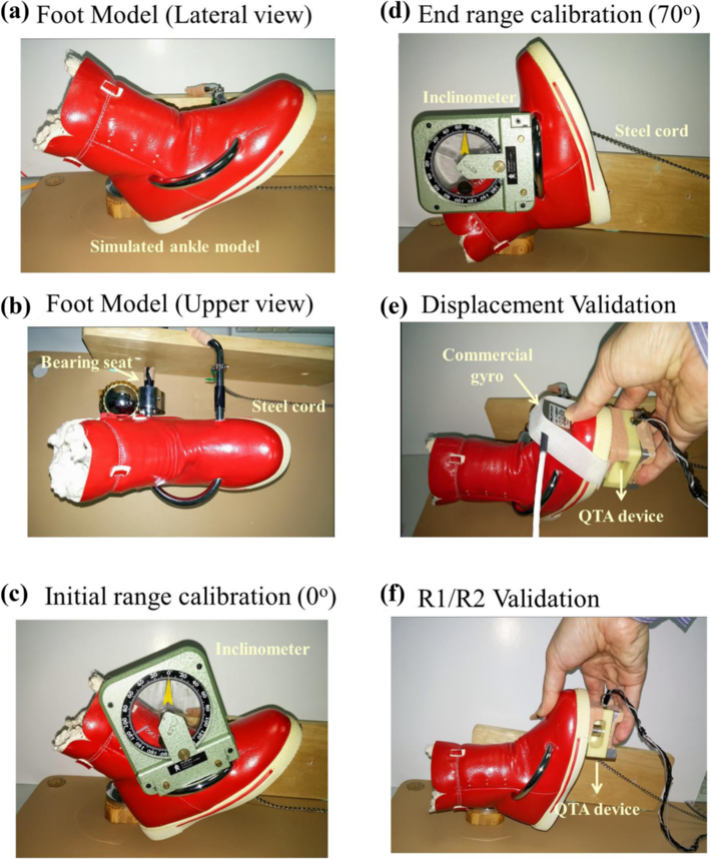
The simulated foot model for validation tests of our measurement system. **a** & **b** The simulated model was designed to rotate around the bearing seat. **c** & **d** The movable range was set and calibrated as 0° to 70°, in a range similar to the real foot available range. The resting position (about 40° elevation from the platform) is defined as 0° of the model. **e** We attached a commercial gyro along with our device to record the angular velocity simultaneously. **f** Test of the feasibility of R1 and R2 estimation by the displacement and resistance signals of QMTA device

Before we applied the QMTA for clinical patients, the performance of the measurement system was first validated. For convenience, we made a simulated foot model with a movable joint to do all the validation tests (as shown in Fig. 3). The simulated model was made by a child shoe filled with casting to simulate the weight of real child foot. A shaft was mounted on the rotation axis of the model and was connected to the platform via a bearing seat. The movable range of the foot model was 0°~70° (assumed maximal movable range of CP subjects) and the end range (70°) is limited by a steel cord. The range was calibrated with a general inclinometer before all the validation tests (Fig. 3(c)&(d)).

To validate the performance of our gyro chip module in the measurement of angular velocity and displacement during stretches, we used a commercial gyro product (Range: +/−600°/s, 2.7x2.4 *x*2.4 cm; Model 11206 AC, Summit Co., Ltd) for comparison. As shown in Fig. 3(e), the examiner held the sensing device (along with commercial gyro) and stretched the foot model under two self-selected speeds (slow and fast). The examiner was instructed to move the model from the movable range 0° to 70° and to cease the stretch when he felt resistance. Four to-and-fro stretches for each speed were performed continuously and the signal of both gyros were recorded and analyzed after. Both gyro signals were filtered (15Hz low-pass filter) and integrated (trapezoid approach) to obtain the displacement information with Matlab software. Both the angular velocity and displacement were compared between two gyros with the correlation coefficient. The averaged error percentage was also calculated for angular displacement to compare the performance of displacement measurement. The error percentage (EP) of the gyro module at each data point was defined as:

1 $$ \mathrm{E}\mathrm{P}\left(\%\right) = \left({\mathrm{D}}_{\mathrm{Gyro}1}\hbox{--}\ {\mathrm{D}}_{\mathrm{Gyro}2}\right)/{\mathrm{D}}_{\mathrm{Gyro}2}*\ 100 $$

where D_Gyro1_ and D_Gyro2_ represents the angular displacement of QMTA device and commercial gyro at one point of time respectively.

The same foot model was used to test the feasibility of R1 and R2 estimation by the displacement and resistance signals of the QMTA device (Fig. 3(f)). The foot model was stretched toward the end-range of 70° from 0° with both slow and fast stretches. The examiner was instructed to move the model five times for each velocity and to stop each stretch when he felt the resistance. Both gyro and resistance signals were filtered (15Hz low-pass filter) and the filtered gyro signal was integrated (trapezoid approach) to get the displacement data. As shown in Fig. 1(c) and (d), peak of resistance was marked and was used to find the resistance angles (R1 and R2) from the derived displacement. For a mechanical joint model, the approximation of static angle R1 and dynamic angle R2 was assumed.

### Subjects recruitment for clinical application

We used our QMTA device and approach to evaluate the effects of BoNT-A injection on spastic calf muscles. Four children with cerebral palsy (CP) were recruited in our study. All subjects were diagnosed and evaluated by a qualified physiatrist before participation. The following procedure of QMTA evaluation was also done by the same physiatrist. The parents of subjects were told the procedures and the necessary information of the experiment. They signed the informed consent before entering the study. The study protocol was approved by the local Ethics Committee and Institutional Review Board (reference number EMRP04098N). All subjects were diagnosed as spastic type CP with involvement of one or both lower limbs. The subjects did not receive leg surgeries before and during the experimental period. All recruited subjects cooperated well during the injection and the evaluation period. OnabotulinumtoxinA (Botox Allergan, CA) injections were performed smoothly for all patients, with the total dosage of 15 unit/kg. The detailed information of subjects is summarized in Table 1.

**Table 1:** Profile of participants

Subject no.	Sex	Diagnosis	GMFCS	Spastic leg	Age (y/o)	Height (cm)	Weight (kg)	Initial angle (R/L) (^o^)
S1	Male	Spastic diplegic CP	III	R/L	8	110	22	40/40
S2	Female	Spastic diplegic CP	III	R/L	5	114	12	40/40
S3	Male	Spastic triplegic CP	III	R/L	7	114	26	40/50
S4	Female	Spastic hemiplegic CP	II	R	6	100	18	40

*CP* Cerebral Palsy, *GMFCS* Gross Motor Function Classification System, *R/L* the right and left side

The examiner was trained to use the device and his stretch maneuvers were validated before evaluation. The examiner was instructed to stretch the foot with usual manner of MTS and to cease the stretch when he felt the resistance of end range. For the clinical evaluation setup, the foot was first placed on a wedge to set the initial stretch angle 40° or 50° of plantar flexion from neutral position (Fig. 2(a)). The examiner first stretched the spastic ankle with slow-velocity and then stretched with fast-velocity to avoid the effects of overactive stretch reflexes. At least six stretches for each velocity were made during an evaluation course. To provide a consistent initial position of ankle joint to minimize the error of displacement due to drift, at least 3 s was reserved between each stretch cycle to ensure the gyroscope signal had returned to a stable baseline. Any stretch that did not follow the above instructions was considered invalid and was discarded online. Since the evaluation was done in the outpatient setting, the entire evaluation process took less than ten minutes for each patient. Only five of six stretches were selected to further process with the same method as described previously.

All subjects were evaluated on the day before BoNT-A injection and four weeks after injection when he/she returned to the outpatient department. The modified Ashworth scale (MAS 0–5) of the tested limb was also collected. Routine rehabilitation program including physical and occupational therapy continued during the four-week experimental period.

### Data analysis

The recorded velocity and resistance signals were offline processed with Matlab program. With derived displacement and resistance during one stretch cycle, the peak of resistance was found and the corresponding displacement angle was considered as the R1 or R2 angle for fast and slow stretch respectively. For analysis of BoNT-A effects on spasticity of calf muscle, we compared MAS, R1 angle, R2 angle, and difference between R2 and R1 angle at baseline and four-week after injection with Wilcoxson signed-rank test. To understand the stretch manners of QMTA evaluation, peak velocities of both slow and fast stretches were also compared with Wilcoxson signed-rank test.

## Results

### Comparison with commercial product

**Fig. 4:**
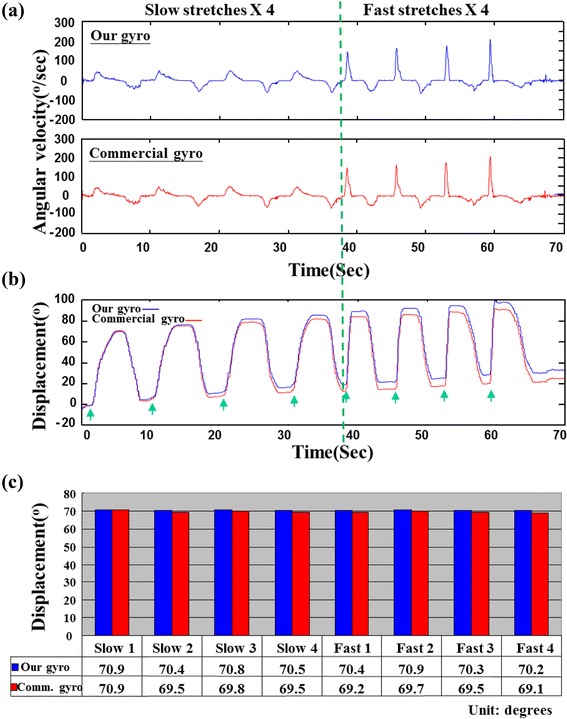
Gyro performance of displacement measurement between our device and commercial product (comm. gyro). **a** Angular velocity signals (with 15Hz low-pass filtering) during four slow and four fast stretches. The signals were almost identical with very good correlation (CC = 0.9957). **b** The derived displacement signals both showed a drift phenomenon (an upward trend was noted). The green arrows indicated the start position of each stretch and were used as a reference to calculate each stretch displacement. **c** For all stretch cycles, the displacement from our gyro was very close to the commercial gyro with a maximal error percentage of 1.7 %

As shown in Fig. 4(a), angular velocity signals from our device and commercial gyro showed similar results with a correlation coefficient of 0.9957. When we derived the angular displacement of both gyros by integration, both results revealed the drift effect as time proceeded (Fig. 4(b)). If we use the commercial gyro as the reference, the averaged error percentage during the 70-s test period was 4.36 %. However, if we calculated the displacement of each stretch individually, the error percentage of measured displacement ranged from 0 % to 1.73 % for all stretch trials which is a direct result of the noisy signal.

### R1 and R2 measurements with foot model

**Fig. 5:**
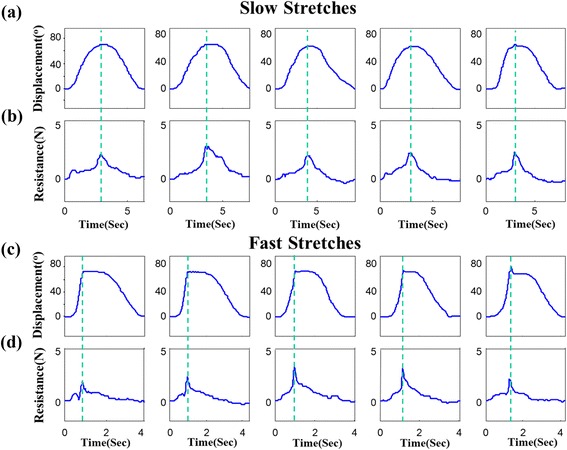
Displacement and resistance curves of foot model testing with five slow and fast stretches. The green dashed lines indicate the peak of resistance curve and were used to find the parameters of R1 and R2. Displacement of joint model for slow and fast stretches are shown in (**a**) and (**c**) respectively. Corresponding resistance curves for slow and fast stretches are shown in (**b**) and (**d**) respectively

Displacement and resistance curves during five slow and fast stretches performed on the simulated joint model are shown in Fig. 5. The parameters R1 and R2 of displacement were derived according to the peak of corresponding resistance in each stretch (green dashed lines in Fig. 5). In this validation trial, the averaged peak velocities of slow and fast stretches were 240.6° /s and 51.7° /s respectively. The averaged R1 and R2 parameters for slow and fast stretches were 70.6° and 69.9° respectively. Finally, the average R2-R1 was −0.6°. For a mechanical joint model, the approximation of static angle R1 and dynamic angle R2 was confirmed.

### Effect of BoNT-A injection

Table 2 shows the outcome measures before and 4 weeks after administration of BoNT-A. Evaluation of spasticity by MAS revealed that there was one grade of improvement in participants 1, 3 and 4; there was a two-grade improvement in participant 2. MAS improved significantly after BoNT-A injection. The mean of R1 did not differ significantly after BoNT-A injection. Improvement of 7.4° was found for the mean of R2 after injection. The angle of R2-R1 also increased significantly after injection.

**Table 2:** Changes of MAS and QMTA parameters after four weeks of BoNT-A injection from seven spastic calf muscles of 4 subjects with CP

Subject	R/L	Weeks	MAS	aR1	aR2	aR2-aR1
S1	R	0	4	35.2	48.8	13.6
		4	3	34.9	55.1	20.2
	L	0	4	42.3	55.7	13.4
		4	3	32.9	54.0	21.1
S2	R	0	4	45.5	59.4	14.0
		4	2	53.8	68.8	15.0
	L	0	4	47.0	60.3	13.3
		4	2	52.7	78.1	25.4
S3	R	0	4	33.5	53.7	20.2
		4	3	46.0	60.9	14.9
	L	0	3	47.8	58.3	10.5
		4	2	47.5	61.1	13.7
S4	R	0	4	32.4	44.1	11.7
		4	3	35.6	54.4	18.8
Week 0	Mean ± SD		3.9 ± 0.4	40.5 ± 6.7	54.4 ± 6.0	13.8 ± 3.1
Week 4			2.6 ± 0.5	43.3 ± 8.8	61.8 ± 8.9	18.4 ± 4.2
	*P* value		<0.001*	0.169	0.009*	0.036*

*MAS* Modified Ashworth Scale, *QMTA* Quantitative Modified Tardieu Approach, *CP* Cerebral Palsy, *R/L* the right and left side, *aR1* averaged R1, *aR2* averaged R2, *aR2-aR1* averaged R2 minus averaged R1, *SD* Standard Deviation

## Discussion

In the study, we have developed a miniature device that can be used as a quantitative measurement for spasticity of calf muscle in children with CP. This novel device along with a miniature gyro sensor and a load cell has the capacity to record joint displacement and resistance by routine clinical approaches and can be used for the evaluation of spasticity. As shown in Fig. 1(b), the portable device can be used in conjunction with the MTS approach. With the data of joint displacement and resistance, resistance angles can be derived to retrieve R1, R2, and the difference between R2 and R1 of spastic muscles in a more objective manner. The stretch maneuver of our quantitative modified Tardieu approach (QMTA) follows the same maneuver of modified Tardieu scale used in clinic. The stop points of R1 and R2 is decided by the same evaluator when he feels the resistance. The validity and reliability of MTS had been established in previous studies [14, 20]. Under this circumstance, our device only needs to evaluate its accuracy in measurement of the joint angles. Validation test of the device showed good performance in displacement measurement (Fig. 4) and resistance angle (Fig. 5). The problem of the drift phenomenon in measurement of joint displacement with gyroscope sensor [28] can be minimized if we keep the gyro signal stable before each stretch and carefully choose the starting position of each stretch (Fig. 4(c)). The results of our validation suggested that our QMTA might be feasible to quantitatively measure the response of BoNT-A injection on spastic calf muscle of children with CP. Compared to previous studies using inertial sensors to determine the catch angles during stretches [22–25], our approach used fewer constraints such as orthoses or fixators on patients and therefore had better compliance. In a clinical setting, less interference on the subject might be necessary. The advantage of our QMTA device over the clinical scales includes its convenience for clinician to use at bed-side and in clinic to objectively investigate the effects and the optimal dosage of BoNT for patients with cerebral palsy.

The MTS used R2, R1, and R2-R1 to measure the different components of spasticity in joints of children with CP. Boyd et al. found a larger value of R2 minus R1 denotes more dynamic component which is more likely to respond to the injection of BoNT-A [14]. Small differences between R2 and R1 implies that more contributions are coming from non-neural biomechanical components, such as intrinsic stiffness. Slow velocity examination of R2 through passive joint ROM indicates the muscle length controlled by neural and non-neural components. Our results showed significant improvements for the difference between R2 and R1 after BoNT administration. This suggests that spasticity was reduced at 4 weeks after injection and this corresponded to the neurophysiological response of treatment with BoNT occurring within four weeks [29]. R2 increased significantly at 4 weeks following BoNT injection in our study. This was compatible with the findings that Park and Kown had observed, which showed decreased muscle spasticity and decreased muscle stiffness at 4 weeks after BoNT injection and intensive rehabilitation treatment which was confirmed by a decrease in the color-coded scale and an increase in the strain ratio using dynamic sonoelastography [30]. Park and Kwon used modified Ashworth scale (MAS) and real-time sonoelastography (RTS) in their study to investigate the neural component and the biomechanical component such as intrinsic stiffness of gastrocnemius muscles after rehabilitation therapy with botulinum toxin A injection in spastic cerebral palsy [30]. Their results demonstrated decreased MAS score and reduced muscle stiffness through RTS at 4 weeks after the intervention. It means that both the neural component and the biomechanical (non-neural) component improved after treatment with simultaneous rehabilitation therapy and botulinum toxin A injection. We assessed the intrinsic muscle stiffness of gastrocnemius muscle by R2 changes in MTS after our intervention. Our participants also received rehabilitation therapy after administration with botulinum toxin A. As a result, the findings from their study were consistent with our results and supported similar conclusions. However, Alhusaini et al. [31] found increased ankle ROM but unchanged passive intrinsic muscle stiffness during low-velocity stretches of the gastrocnemius in 16 children with CP at 6 weeks after BoNT-A injection. As the maximal response occurs within 4 weeks after administration of BoNT-A [32], the different time point of follow-up may account for various effects in above studies.

Some trials found that BoNT-A injection and other interventions such as serial casting and stretching might improve the non-neural components of hypertonia. Besides, contradictory results exist for ROM gained in ankle after administration with BoNT-A. In the assessment of protocol for serial casting after botulinum toxin injections to treat equinus in children with CP, Kelly et al. found that improvements in the MTS were seen in R1 and R2 values for dorsiflexion with both the knee in extension and in flexion [33]. Their findings indicated that both R1 and R2 could improve after administration of BoNT-A and other intervention. Koman et al. conducted a multi-center trial of 207 children with CP having BoNT-A injections to the gastrocnemius muscles, they found the active movement of ankle improved after injection [34]. All the participants in our study received routine rehabilitation programs such as strengthening and ankle stretching after BoNT-A injection, with each patient performing three, 30 min sessions per week for a total of 4 weeks. Therefore, our findings of improvements of R2-R1, R2 and R1 are compatible with the results of above studies.

On the contrary, previous studies have shown that the effects of BoNT-A injection on ROM had a short duration and contractures continued to develop despite the muscle tone had decreased after injection. The prospective long-term follow-up investigation by Tedroff et al. demonstrated an increase of 4° to 7° in dorsiflexion ROM in the calf of children with CP after BoNT injection [35]. However, the ROM decreased as soft tissues reverted to their original status. As the participants were only assessed at 4 weeks after injection in our study, we may have failed to find the changes after 4 weeks in these patients. This may explain the differences of our findings as compared to the results of Alhusaini et al. that was followed before and at 6 weeks after injection. Some histological changes in the muscles had been reported after BoNT-A injection in their study. Dodd et al. found a shift in heavy-chain composition from faster to slower isoforms in rat muscle after injection with BoNT-A [36]. Legerlotz et al. also noted that BoNT-A injection resulted in slower myosin heavy-chain isoform composition and reduced titin content by 18 % in the gastrocnemius muscles of juvenile rats [37]. These reactions after BoNT administration may change the characteristics of the muscle that account for what we saw in our results. Further studies are necessary to illuminate the role of BoNT-A in neural and non-neural components of patients with spasticity.

The physician involved in the assessment of angles via QMTA in this study was satisfied with the tiny, convenient and quick features of this device. The entire evaluation process took less than ten minutes for each patient. It is suitable for the routine assessment of spasticity in the outpatient setting, especially for the children clients. In the double-blind study investigating the effect of BoNT-A, Sutherland et al. suggested that children under 4 years of age may have difficulty in cooperation during the evaluations [38].

There are several limitations in our present pilot study. First, we did not collect the EMG data from our subjects during stretches. EMG can be used to monitor the relaxation of stretched muscles [39] and to confirm the presence of stretch reflexes during fast stretches [40]. It may help the clinician decide the points of R1 and R2 more precisely. As our study was performed in the clinical setting of an outpatient department, we tried to minimize the time spent in the evaluation by omitting other interventions such as EMG, to obtain a better compliance of children with CP. In total, we spent less than 10 min for QMTA evaluation of both legs in each CP subject, which included communication, waiting and the examiner completing the steps of QMTA we mentioned in section 2. Second, the sample size of subjects was very small in this pilot study. More participants are necessary for future studies. Furthermore, the follow-up period should be longer to follow the long-term effect of BoNT-A on neural and non-neural components of spastic calf muscle.

## Conclusions

Our quantitative Tardieu scale approach (QMTA) is feasible to quantitatively measure the effect of botulinum toxin injection for gastrocnemius muscle spasticity in children with cerebral palsy. It may help clinicians decide the optimal dosage of botulinum toxin type A for spasticity of calf muscle in children with CP. This new device needs more participants and longer follow-up periods in future studies.
